# Supplementary material for: Operational indicators for pulmonary tuberculosis diagnosis in people living with HIV before and after Xpert MTB/RIF implementation in the state of São Paulo, Brazil
Source: PLoS One. 2024 Jun 7;19(6):e0305063. doi: 10.1371/journal.pone.0305063 (PMC11161080; doi:10.1371/journal.pone.0305063)
Supplement: Attachment — Submitted filename: Response to Reviewers.docx [file pone.0305063.s003.docx]

Dear editor,

Thank you for giving us the opportunity to submit a revised draft of the manuscript “Operational indicators for pulmonary tuberculosis diagnosis in people living with HIV before and after Xpert MTB/RIF implementation in the state of São Paulo, Brazil” for publication in the Plos One. We appreciate the time and effort that you and the reviewers dedicated to providing feedback on our manuscript and are grateful for the insightful comments on and valuable improvements to our paper.

We have incorporated most of the suggestions made by the reviewers. Those changes are highlighted within the manuscript. Please see below, a point-by-point response to the reviewers’ comments and concerns.

**Journal Requirements:**

**PONE-D-23-42570**

1. OK

2.OK

3. The data were obtained after receiving acceptance by the Research Ethics Committee. As this is anonymous data, there was no need for a Informed Consent Form from the study sample. The term "anonymously" was included in the methods section, as well as indicating that the study did not address people under 18 years of age.

4,5,6. This study was financed by the Coordenação de Aperfeiçoamento de Pessoal de Nível Superior – Brasil (CAPES) [Financial Code 001] (PhD scholarship), Conselho Nacional de Desenvolvimento Científico e Tecnológico (CNPq) – Produtivity in Research Sponsorship [grant number 317170-2021-0] (advisor's scholarship) and Fundação de Amparo à Pesquisa do Estado de São Paulo [process number 2022/00025-2] (research fund to present results in scientific events).

7. A complementary file with the data was shared with the manuscript.

**Reviewer’s comments:**

**01. Reviewer #1:** We believe that the modifications made in the results session of this study gave greater solidity to the conclusions;

**02. Reviewer #1 and 2:** The analyzes were revised based on the comments presented by the reviewers, making them more robust

**03.** The authors make the data available

**04. Reviewer #1:** The text has been revised and presented appropriately

**05**. **Reviewer #1-** We believe that after the results revision, the conclusions became more suitable, which were also reviewed.

**Minor comments:**

1.The review’s conclusion was in the text: a literature review in December 2019 highlighted a higher accuracy of Xpert in confirming pulmonary TB (PTB) in PLHIV compared to smear microscopy, and showed similar performance to culture and much faster

**2.** Lines- 113-116 were corrected

**3.** As this is secondary data, it was not possible to obtain information on the amount of substance use. The appropriate term to name it was included in the table and text - use of psychoactive substances.

**4.** Lines-131-135 and 158-172 were corrected.

**5.** Figura 1 was translated.

**6**. We did not insert any explanation in Figure 2 legend, cause we think they are mentioned in the text.

**7**. Between lines 303-307, we have already showed possible explanations for the underuse of RMT-TB for diagnosing tuberculosis.

**8**. The text was reviewed.

**Major comments:**

**1.** This study was developed based on the Strengthening the Reporting of Observational Studies in Epidemiology (STROBE) initiative checklist [20] and the suggested additions to the STROBE document for ecologic reporting [21].

**2.** p-value was included on tables 1, 2 and 3.

**3**. The authors carried out the suggested reading regarding the recommendations on writing Race/Ethnicity, adjusting the text as requested. However, the “Brown” race is a Brazilian term, therefore, we did not find a better way to name it.

**4**.We agree with the suggestion and included it on conclusions.

**6**. No. We do not want to publish our full peer review.
